# First principles and mean field study on the magnetocaloric effect of YFe_3_ and HoFe_3_ compounds

**DOI:** 10.1038/s41598-023-29676-9

**Published:** 2023-02-18

**Authors:** Mohammed Said Mohammed Abu-Elmagd, Tarek Hammad, Ahmed Abdel-Kader, Nesreen El-Shamy, Sherif Yehia, Samy H. Aly, Fatema Z. Mohammad

**Affiliations:** 1 Department of Engineering Mathematics and Physics, Higher Institute of Engineering, El Shourok Academy, Cairo, Egypt; 2 Department of Physics, Faculty of Science, Helwan University, Cairo, Egypt; 3 Physics Department, Faculty of Science, Taibah University, Madinah, Saudi Arabia; 4 Department of Physics, Faculty of Women, Ain Shams University, Cairo, Egypt; 5 Department of Physics, Faculty of Science, Damietta University, New Damietta, Egypt

**Keywords:** Electronic properties and materials, Magnetic properties and materials, Condensed-matter physics, Theory and computation, Computational methods, Electronic structure

## Abstract

In this work, the magnetothermal characteristics and magnetocaloric effect in YFe_3_ and HoFe_3_ compounds are calculated as function of temperature and magnetic field. These properties were investigated using the two-sublattice mean field model and the first-principles DFT calculation using the WIEN2k code. The two-sublattice model of the mean-field theory was used to calculate the temperature and field-dependences of magnetization, magnetic heat capacity, magnetic entropy, and the isothermal change in entropy ∆S_m_. We used the WIEN2k code to determine the elastic constants and, subsequently, the bulk and shear moduli, the Debye temperature, and the density-of-states at E_f_. According to the Hill prediction, YFe_3_ has bulk and shear moduli of roughly 99.3 and 101.2 GPa respectively. The Debye temperature is ≈ 500 K, and the average sound speed is ≈ 4167 m/s. In fields up to 60 kOe and at temperatures up to and above the Curie point for both substances, the trapezoidal method was used to determine ∆S_m_. For instance, the highest ∆S_m_ values for YFe_3_ and HoFe_3_ in 30 kOe are approximately 0.8 and 0.12 J/mol. K, respectively. For the Y and Ho systems, respectively, the adiabatic temperature change in a 3 T field decreases at a rate of around 1.3 and 0.4 K/T. The ferro (or ferrimagnetic) to paramagnetic phase change in these two compounds, as indicated by the temperature and field dependences of the magnetothermal and magnetocaloric properties, ∆S_m_ and ∆T_ad_, is a second-order phase transition. The Arrott plots and the universal curve for YFe_3_ were also calculated and their features give an additional support to the second order nature of the phase transition.

## Introduction

The magneto-caloric effect (MCE) is a well-known interesting phenomenon that involves a change in the temperature upon applying/removing an external magnetic field. Its physics and applications are of continuous interest to researchers worldwide. [e.g.,^1,2^].

Some of the intermetallic compounds that exist in the rare-earth-iron system are, for example, RFe_2_, RFe_3_, R_6_Fe_23_ and R_2_Fe_17_^3–5^. Much work has been dedicated to the magnetic properties of those compounds. The RT_2_ compounds, where T = Fe, Co, have been also investigated^6–9^. Roe et al.^10^ determined the magnetic structure of the iron-holmium binary system. Hoffer and Salmans^11^ measured the magnetization of the RFe_3_ compounds as function of temperature. On the application side, magnetic refrigerators, in particular those operating close to room temperature are a subject of extensive research. One of the reasons of such interest in the magnetocaloric-based refrigerators are the relatively higher efficiency and the lower impact on environment as compared to the conventional ones^12,13^. Rare-earth intermetallic compounds were a subject of many works due to their distinguished electronic, magnetic and magnetocaloric properties^14–18^. The RFe_3_ compounds, in particular, are of interest for both their magnetostrictive and magnetic properties^19–21^, however not much investigations were done on their magnetocaloric properties.

Our motivation, in this paper, is studying the magnetothermal and the magnetocaloric effect in YFe_3_ and HoFe_3_, using both ab initio calculation and the molecular field theory, in the two-sublattice model^22–24^. We report on both temperature and magnetic field dependences of magnetization, magnetic specific heat magnetic entropy, isothermal change in entropy and adiabatic change in temperature in the temperature interval of 0–800 K. We report on new calculation of the elastic constants, the bulk, the shear moduli and the electronic and lattice contributions to the total heat capacity and entropy using the well-known WIEN2k code package^25–29^. Another motivation of the present work is studying the order of the phase transition in these compounds in the light of the dependence of their properties on both temperature and field.

To calculate the elastic moduli of YFe_3_ we used the linear augmented plane-wave (LAPW) method by WIEN2K code^30^. This Package uses the energy approach calculation ^31^ that calculates the elastic constants by second-order derivative. The results of these calculations are then used to calculate the Debye temperature and the electronic heat capacity coefficient for ${\text{ YFe}}_{3}$. These two parameters for HoFe_3_ are obtained from available published data (Persson, the materials project)^32^.

## Computational methods

### DFT

The Wien2K electronic structure code is based on the Density Functional Theory (DFT)^33^. It employs the Full-Potential Linearized Augmented Plane Wave (FPLAPW)^34^. For correlation and exchange potentials, the Wien2k code^30^ employs the Local Density Approximation (LDA) of Perdew and Wang^35^ and the Generalized Gradient Approximation (GGA) of Perdew, Burke, and Ernzerhof^36^. Core and valence states are calculated in a self-consistent manner. For the spherical part of the potential, the core states are treated fully relativistically, while the valence states are treated using the full potential. To reduce linearization errors in R and Fe spheres, local orbital extensions^37^ with a converged basis of approximately 1000 basis functions are used. For the self-consistent band structure calculations, we used the modified tetrahedron method for Brillouin zone integration. Self-consistent calculations were performed, and convergence was checked by varying the number of k points in the irreducible Brillouin zone up to 64 points The ab initio calculated elastic properties of different systems have been reported, e.g. for YFe_5_^38^, PrX_2_ (X = Fe, Mn, Co) compounds^39^, Cr-based full-Heusler alloys^40^ and GdFe_2_^41^. Also ab initio calculation on the effect of applying pressure on PtXSb and GdN systems were reported, respectively by Habbak et al.^42^ and Reham Shabara et al.^43^. Magnetic properties and electronic structure of ThCo_4_B were reported by Abu-Elmagd et al.^44^ and Sherif Yehia et al.^45^ have reported on the spin and charge density maps of SmCo_5_.

### Elastic properties

The Lagrangian theory of elasticity is used to describe the elastic properties. According to this theory, a solid is a homogeneous and anisotropic elastic medium. As a result, strains (defined as the fractional change in length) are homogeneous and can be represented using symmetric second rank tensors.

1 $$\partial_{ij} = \varepsilon_{ij} + \frac{1}{2}\mathop \sum \limits_{k} \varepsilon_{ik} \varepsilon_{kj}$$

where ∂ is the Lagrangian strain and ε_*ij*_ are homogeneous strain parameters and i and j indicate Cartesian components^44^.

When we cast a crystal structure's Bravais lattice vectors, under an isotropic pressure, in a matrix form (R), the small homogeneous deformation (strain) distorts the Bravais lattice vectors of this crystal and hence cause a distortion of the lattice (R’) expressed by multiplying with a symmetric deformation matrix i.e. (R’ = R*D), where D is:

2 $$D = I + \varepsilon = \left( {\begin{array}{*{20}c} {1 + \varepsilon_{11} } & {\varepsilon_{12} } & {\varepsilon_{13} } \\ {\varepsilon_{21} } & {1 + \varepsilon_{22} } & {\varepsilon_{23} } \\ {\varepsilon_{31} } & {\varepsilon_{32} } & {1 + \varepsilon_{33} } \\ \end{array} } \right)$$

where I is a unique matrix that represents the symmetric strain tensor.

Now, we express the total energy of a crystal (R’), under strain in terms of a power series of the Lagrangian strain (∂):

3 $$E\left( {\partial ,V} \right) = E_{0} + V_{0} \mathop \sum \limits_{ij} \left( {\tau_{ij} \partial_{ij} } \right) + \frac{{V_{0} }}{2}\mathop \sum \limits_{ijkl} \left( {C_{ijkl} \partial_{ij} \partial_{kl} } \right)$$

where E (E_0_) is the energy and V (V_0_) is the volume of strained system.

The current ${\text{RFe}}_{3}$ compounds (R = rare earth) crystallize in the ${\text{PuNi}}_{3}$-type structure, with space group 194-P63/mmc (Hexagonal).

C_11_, C_12_, C_13_, C_33_, and C_55_ are the five independent elastic constants for a hexagonal symmetry. We need five different strains to determine these elastic constants because we have five independent elastic constants. The five distortions are discussed further below. The first distortion^46^ is as follows:

4 $$\left( {\begin{array}{*{20}c} {1 + \delta } & 0 & 0 \\ 0 & {1 + \delta } & 0 \\ 0 & 0 & 1 \\ \end{array} } \right)$$

and it modifies the basal plane while keeping the z-axis constant. As a result, the symmetry of the strained lattice remains hexagonal, and the energy for this distortion can be calculated as follows:

5 $$E\left( {V,\delta } \right) = E\left( {V_{0} ,0} \right) + V_{0} \delta \left( {\tau_{1} + \tau_{2} } \right) + V_{0} \left[ {\left( {C_{11} + C_{12} } \right)\delta^{2} + O\left( {\delta^{3} } \right)} \right]$$

The second type of distortion is a volume-conserved distortion and lead to orthorhombic symmetry and written as:

6 $$\left( {\begin{array}{*{20}c} {\left( {\frac{1 + \delta }{{1 - \delta }}} \right)^{1/2} } & 0 & 0 \\ 0 & {\left( {\frac{1 - \delta }{{1 + \delta }}} \right)^{{^{1/2} }} } & 0 \\ 0 & 0 & 1 \\ \end{array} } \right)$$

and the energy for this distortion can be obtained as:

7 $$E\left( {V,\delta } \right) = E\left( {V_{0} ,0} \right) + V_{0} \left[ {\left( {C_{11} + C_{12} } \right)\delta^{2} + O\left( {\delta^{3} } \right)} \right]$$

The third strain we have used is given by:

8 $$\left( {\begin{array}{*{20}c} 1 & 0 & 0 \\ 0 & 1 & 0 \\ 0 & 0 & {1 + \delta } \\ \end{array} } \right)$$

This strain changes C lattice parameter and keeps the strained lattice's symmetry hexagonal, and the energy for this distortion can be obtained by

9 $$E\left( {V,\delta } \right) = E\left( {V_{0} ,0} \right) + V_{0} \delta \left( {\tau_{3} } \right) + V_{0} \left[ {\left( {C_{33} } \right)\frac{{\delta^{2} }}{2} + O\left( {\delta^{3} } \right)} \right]$$

The fourth elastic constant, C_55_, is determined by a lattice deformation that results in a low-symmetry object. The deformation is written as follows:

10 $$\left( {\begin{array}{*{20}c} 1 & 0 & \delta \\ 0 & 1 & 0 \\ \delta & 0 & 1 \\ \end{array} } \right)$$

and it leads to triclinic symmetry and the energy for this deformation can be written as:

11 $$E\left( {V,\delta } \right) = E\left( {V_{0} ,0} \right) + V_{0} \delta \left( {\tau_{5} } \right) + V_{0} \left[ {\left( {2C_{55} } \right)\delta^{2} + O\left( {\delta^{3} } \right)} \right]$$

Finally, the last strain we have used is volume-conserved, keeps the symmetry of the strained lattice hexagonal, and can be written as:

12 $$\left( {\begin{array}{*{20}c} {\left( {1 + \delta } \right)^{ - 1/3} } & 0 & 0 \\ 0 & {\left( {1 + \delta } \right)^{ - 1/3} } & 0 \\ 0 & 0 & {\left( {1 + \delta } \right)^{2/3} } \\ \end{array} } \right)$$

and the energy for this strain is given by

13 $$E\left( {V,\delta } \right) = E\left( {V_{0} ,0} \right) + V_{0} \left[ {\left( {C_{zz} } \right)\frac{{\delta^{2} }}{9} + O\left( {\delta^{3} } \right)} \right]$$

and

14 $$C_{ZZ} = C_{11} + C_{12} + 2C_{33} - 4C_{13}$$

Isotropic elastic constants such as the bulk, shear, and Young moduli can be determined using appropriate averaging procedures. All the previous parameters including the Young modulus, E, and the Poisson ratio, ν, the bulk modulus, B, and the shear modulus, G are calculated by IRelast Package^47^ as available in the Wien2k Package^48^. As for the crystal structure of the two compounds: YFe_3_ crystallizes in the hexagonal structure with space group P6_3_/mmc and HoFe_3_ crystallizes in the trigonal structure with ${\overline{\text{R}}\text{3m}}$ space group. All the information about the lattice parameters and the locations of the Y or Ho and Fe atoms in the unit cells is available^32^.

### Thermomagnetic properties

The total effective fields of the R and Fe sublattices are expressed as follows by molecular field theory (MFT):

15 $${\overline{\text{H}}}_{{\text{R}}} \left( {\text{T}} \right) = {\overline{\text{H}}} + {\text{d}}\left[ {{\text{n}}_{{{\text{RR}}}} \overline{\mu }_{{\text{R}}} \left( {\text{T}} \right) + 3{\text{n}}_{{{\text{RF}}}} \overline{\mu }_{{\text{F}}} \left( {\text{T}} \right)} \right]$$

16 $${\overline{\text{H}}}_{{\text{F}}} \left( {\text{T}} \right) = {\overline{\text{H}}} + {\text{d}}\left[ {3 {\text{n}}_{{{\text{FF}}}} \overline{\mu }_{{\text{F}}} \left( {\text{T}} \right) + {\text{n}}_{{{\text{RF}}}} \overline{\mu }_{{\text{R}}} \left( {\text{T}} \right)} \right]$$

In these equations ${\overline{\text{H}}}$ represents the applied field, $\overline{\mu }_{{\text{F}}}$ the magnetic moment per Fe ion at temperature T in units of the Bohr magneton ($\mu_{{\text{B}}}$), and $\overline{\mu }_{{\text{R}}}$ the magnetic moment per rare-earth ion. The factor d converts the moment per ${\text{RFe}}_{3}$ unit from $\mu_{{\text{B}}}$ to gauss:

17 $${\text{d}} \equiv \frac{{{\text{N}}_{{\text{A}}} \mu_{{\text{B}}} {\uprho }}}{{\text{A}}}$$

where N_A_ is Avogadro's number, ρ the density in ${\text{g}}/{\text{cm}}^{3}$, and A is the ${\text{RFe}}_{3}$ formula weight in g/mol. With these definitions the fields: ${\overline{\text{H}}}$,${\overline{\text{H}}}_{{\text{R}}}$ and ${\overline{\text{H}}}_{{\text{F}}}$ are specified in gauss, and the molecular field coefficients ${\text{n}}_{{{\text{RR}}}}$,${\text{n}}_{{{\text{RF}}}}$ and ${\text{n}}_{{{\text{FF}}}}$ respectively, describing the R-R, R-Fe, and Fe–Fe magnetic interactions, are dimensionless.

We begin with the magnetic energy of a binary magnetic compound to calculate the magnetic specific heat.

18 $$U = - \frac{1}{2}\left[ {n_{RR} M_{R}^{2} \left( T \right) + 9 n_{FF} M_{F}^{2} \left( T \right) + 6n_{RF} M_{R} \left( T \right)M_{F} \left( T \right)} \right]$$

The magnetic specific heat is calculated as follows:

19 $${\text{C}}_{{\text{m}}} \left( {{\text{T}},{\text{H}}} \right) = \left( {\frac{{\partial {\text{U}}\left( {{\text{T}},{\text{H}}} \right)}}{{\partial {\text{T}}}}} \right)_{H}$$

The magnetic entropy is calculated from the numerical integration of the magnetic heat capacity as follows:

20 $${\text{S}}_{{\text{m}}} \left( {{\text{T}},{\text{H}}} \right) = \mathop \int \limits_{0}^{{\text{T}}} \frac{{{\text{C}}_{{\text{m}}} \left( {{\text{T}},{\text{H}}} \right)}}{{\text{T}}}{\text{dT}}$$

The total heat capacity ${\text{C}}_{{{\text{total}}}}$ is made up of three contributions: the lattice heat capacity ${\text{ C}}_{{\text{l}}}$, the electronic heat capacity ${\text{C}}_{{\text{e}}}$ and the magnetic heat capacity ${\text{ C}}_{{\text{m}}}$:

21 $${\text{C}}_{{{\text{total}}}} = {\text{C}}_{{\text{l}}} + {\text{C}}_{{\text{e}}} + {\text{C}}_{{\text{m}}}$$

The lattice heat capacity is expressed as:

22 $${\text{C}}_{{\text{l}}} = 9{\text{R}}\left[ {4\left( {\frac{{\text{T}}}{{\theta_{{\text{D}}} }}} \right)^{3} \mathop \int \limits_{0}^{{\text{x}}} \frac{{{\text{x}}^{3} }}{{{\text{e}}^{{\text{x}}} - 1}}{\text{dx}} - \frac{{\text{x}}}{{{\text{e}}^{{\text{x}}} - 1}}} \right]$$

where ${\text{x}} = \theta_{{\text{D}}} /{\text{T}}$ and $\theta_{{\text{D}}}$ is Debye temperature, which can be calculated using Eq. (23)^49^

23 $$\theta_{{\text{D}}} = \frac{{\text{h}}}{{{\text{k}}_{{\text{B}}} }}\left[ {\frac{{3{\text{n}}}}{{4{\uppi }}}\left( {\frac{{{\text{N}}_{{\text{A}}} }}{{\text{A}}}\rho } \right)} \right]^{1/3} {\text{v}}_{{\text{m}}}$$

where h is the Planck's constant, ${\text{k}}_{{\text{B}}}$ is the Boltzmann constant,${\text{v}}_{{\text{m}}}$ is the average sound velocity, and n is the number of atoms per formula unit^50^. Equation (24) gives the average sound velocity in a polycrystalline material:

24 $${\text{v}}_{{\text{m}}} = \left[ {\frac{1}{3} \left( {\frac{2}{{{\text{v}}_{{\text{l}}}^{3} }} + \frac{1}{{{\text{v}}_{{\text{s}}}^{3} }}} \right)} \right]^{{ - \frac{1}{3}}}$$

where ${\text{v}}_{{\text{l}}}$ and ${\text{v}}_{{\text{s}}}$ are, respectively, the longitudinal and transverse sound velocities, which can be calculated using the shear and the bulk moduli G and B, from Navier’s equation^51^:

25 $${\text{v}}_{{\text{l}}} = \sqrt {\frac{{B + \frac{4G}{3}}}{\rho }} ,\;{\text{ and}}\; {\text{v}}_{{\text{s}}} = \sqrt {\frac{G}{\rho }}$$

The electronic heat capacity is given by:

26 $${\text{C}}_{{\text{e}}} = \frac{{\pi^{2} {\text{k}}_{{\text{B}}}^{2} {\text{D}}\left( {{\text{E}}_{{\text{F}}} } \right)}}{3}{\text{T}} = \gamma_{{\text{e}}} {\text{T}}$$

where $\gamma_{{\text{e}}} = \frac{{\pi^{2} {\text{k}}_{{\text{B}}}^{2} {\text{D}}\left( {{\text{E}}_{{\text{F}}} } \right)}}{3}$ represents the electronic heat-capacity coefficient, and ${\text{D}}\left( {{\text{E}}_{{\text{F}}} } \right)$ represents the electron density of states at the Fermi energy ${\text{E}}_{{\text{F}}}$.

A magnetic conducting material's total entropy includes three contributions: lattice (${\text{S}}_{{\text{l}}}$), electronic (${\text{S}}_{{{\text{el}}}}$) and magnetic entropy (${\text{S}}_{{\text{m}}}$). The total entropy can be calculated by numerically integrating the total heat capacity:

27 $$S\left( T \right) = \mathop \int \limits_{{T_{1} }}^{{T_{2} }} \frac{{C_{H} \left( T \right)}}{T} {\text{d}}T$$

The lattice entropy is given by:

28 $$S_{l} = 3R\left[ {4\left( {\frac{T}{{\theta_{D} }}} \right)^{3} \mathop \int \limits_{0}^{{\frac{{\theta_{D} }}{T}}} \frac{{x^{3} }}{{e^{x} - 1}} {\text{d}}x - \ln (1 - e^{{ - \frac{{\theta_{D} }}{T}}} )} \right]$$

The electronic entropy is given, like the electronic heat capacity, by Eq. (26).

### Magnetocaloric effect

The MCE is inherent in all magnetic materials and is induced by the magnetic lattice coupling with the applied magnetic field. The MCE is distinguished by two parameters: the adiabatic temperature change ΔT_ad_ (T, ΔH) and the isothermal entropy change $\Delta {\text{S}}_{{\text{m}}}$(T, ΔH). In an isothermal process, the entropy change caused by a magnetic field variation from H_1_ to H_2_, in compounds undergoing a second order phase transition, is calculated using the following Maxwell relation^52^:

29 $$\left( {\frac{{\partial {\text{S}}\left( {{\text{T}},{\text{H}}} \right)}}{{\partial {\text{H}}}}} \right)_{{\text{T}}} = \left( {\frac{{\partial {\text{M}}\left( {{\text{H}},{\text{T}}} \right)}}{{\partial {\text{T}}}}} \right)_{{\text{H}}}$$

30 $$\Delta {\text{S}}_{{\text{m}}} \left( {{\text{T}},\Delta {\text{H}}} \right) = \mathop \int \limits_{{{\text{H}}_{1} }}^{{{\text{H}}_{2} }} \left( {\frac{{\partial {\text{M}}\left( {{\text{H}},{\text{T}}} \right)}}{{\partial {\text{T}}}}} \right)_{{\text{H}}} {\text{dH}}$$

The adiabatic temperature change $\Delta {\text{T}}_{{{\text{ad}}}}$ is calculated from the following equation:

31 $$\Delta {\text{T}}_{{{\text{ad}}}} \left( {{\text{T}},\Delta {\text{H}}} \right) = - \mathop \int \limits_{{{\text{H}}_{1} }}^{{{\text{H}}_{2} }} \frac{{\text{T}}}{{{\text{C}}\left( {{\text{T}},{\text{H}}_{{\text{F}}} } \right)}}\left( {\frac{{\partial {\text{M}}\left( {{\text{H}},{\text{T}}} \right)}}{{\partial {\text{T}}}}} \right)_{{\text{H}}} {\text{dH}}$$

It is clear from Eq. (31) that the total heat capacity is applied field-dependent. However if ${\text{C}}\left( {{\text{T}},{\text{H}}_{{\text{F}}} } \right)$ has a weak field dependence, we can rewrite Eq. (31) as follows^53^:

32 $$\Delta {\text{T}}_{{{\text{ad}}}} \left( {{\text{T}},\Delta {\text{H}}} \right) \approx \frac{{ - {\text{T}}}}{{{\text{C}}\left( {{\text{T}},{\text{H}}_{{\text{F}}} } \right)}}\mathop \int \limits_{{{\text{H}}_{1} }}^{{{\text{H}}_{2} }} \left( {\frac{{\partial {\text{M}}\left( {{\text{H}},{\text{T}}} \right)}}{{\partial {\text{T}}}}} \right)_{{\text{H}}} {\text{dH}}$$

## Results and discussion

### Density of states

**Figure 1:**
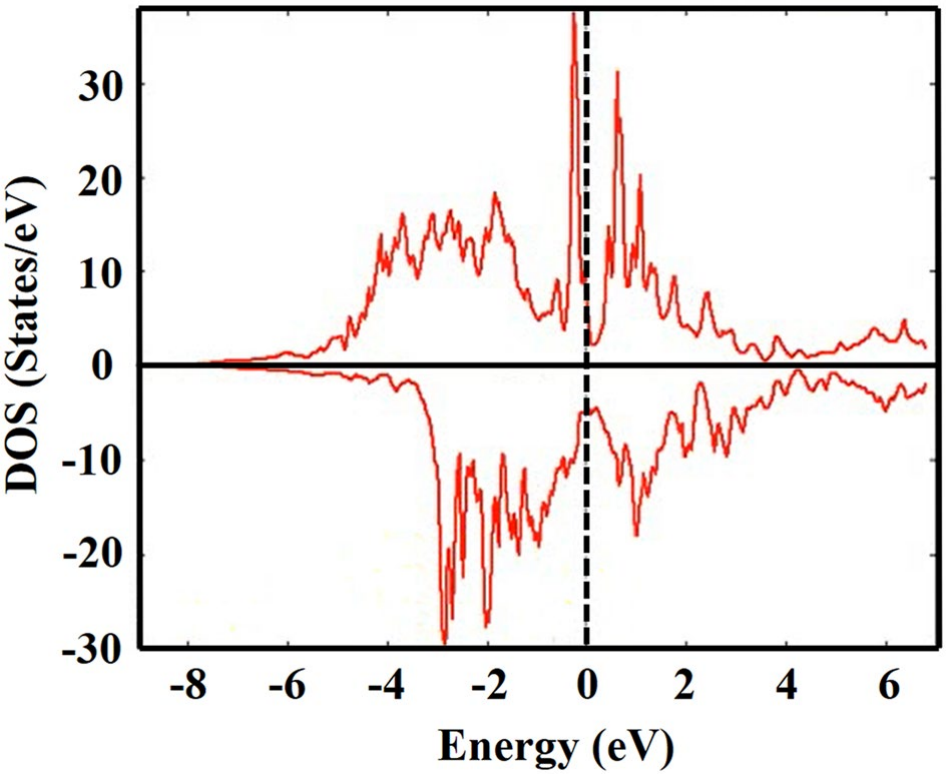
The spin-up and spin-down electronic density of states (DOS) for YFe_3_.

**Figure 2:**
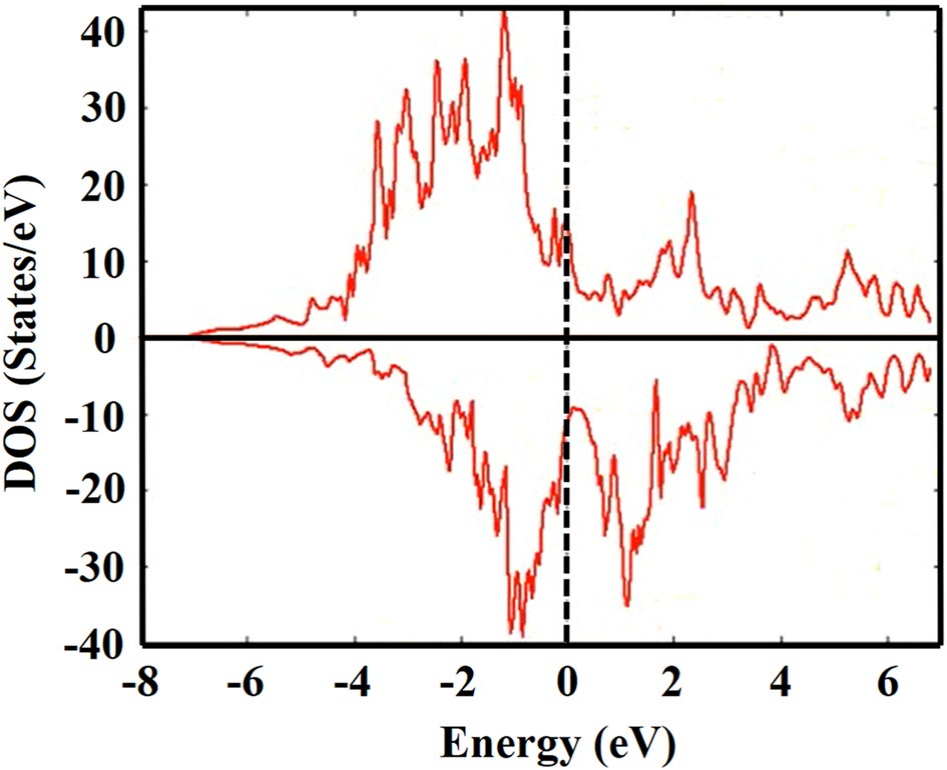
The spin-up and spin-down electronic density of states (DOS) for HoFe_3_.

The Wien2K electronic structure code calculated the total DOS (density of states) for YFe_3_ and HoFe_3_ as shown in Figs. 1 and 2 respectively.

The total DOS, at ${\text{E}}_{{\text{f}}}$ for these two compounds are 10.915 and 24.461 states/eV, respectively. Therefore the electronic heat capacity coefficients, as calculated from Eq. 26, are 0.0256 and 0.0575 J/mol. K^2^ respectively. The DOS of both compounds show that these two systems are metallic with fairly large density of states, at Fermi energy, in both the spin up and spin down configuration. This, of course, demands taking the electronic contribution to the heat capacity into consideration as we pointed out (Eq. 26).

### Magnetization

**Figure 3:**
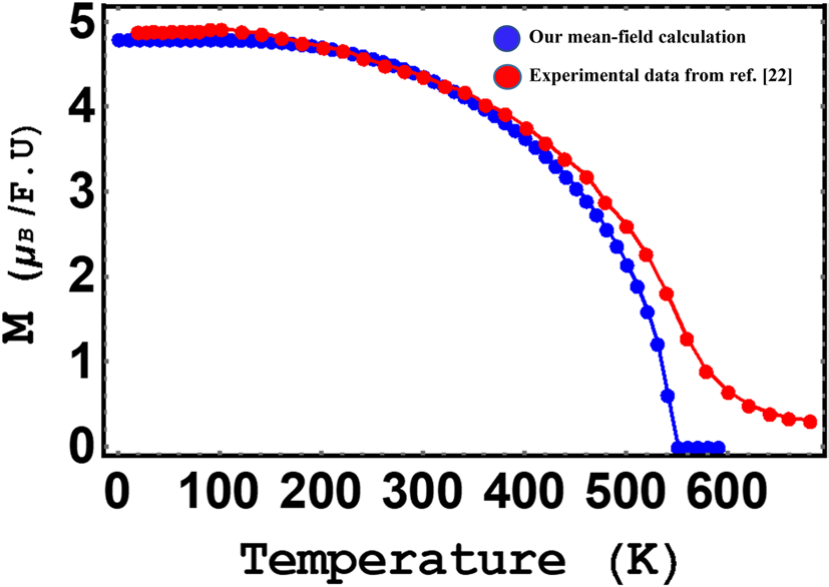
Total magnetization of YFe_3_ in zero field vs. temperature.

**Figure 4:**
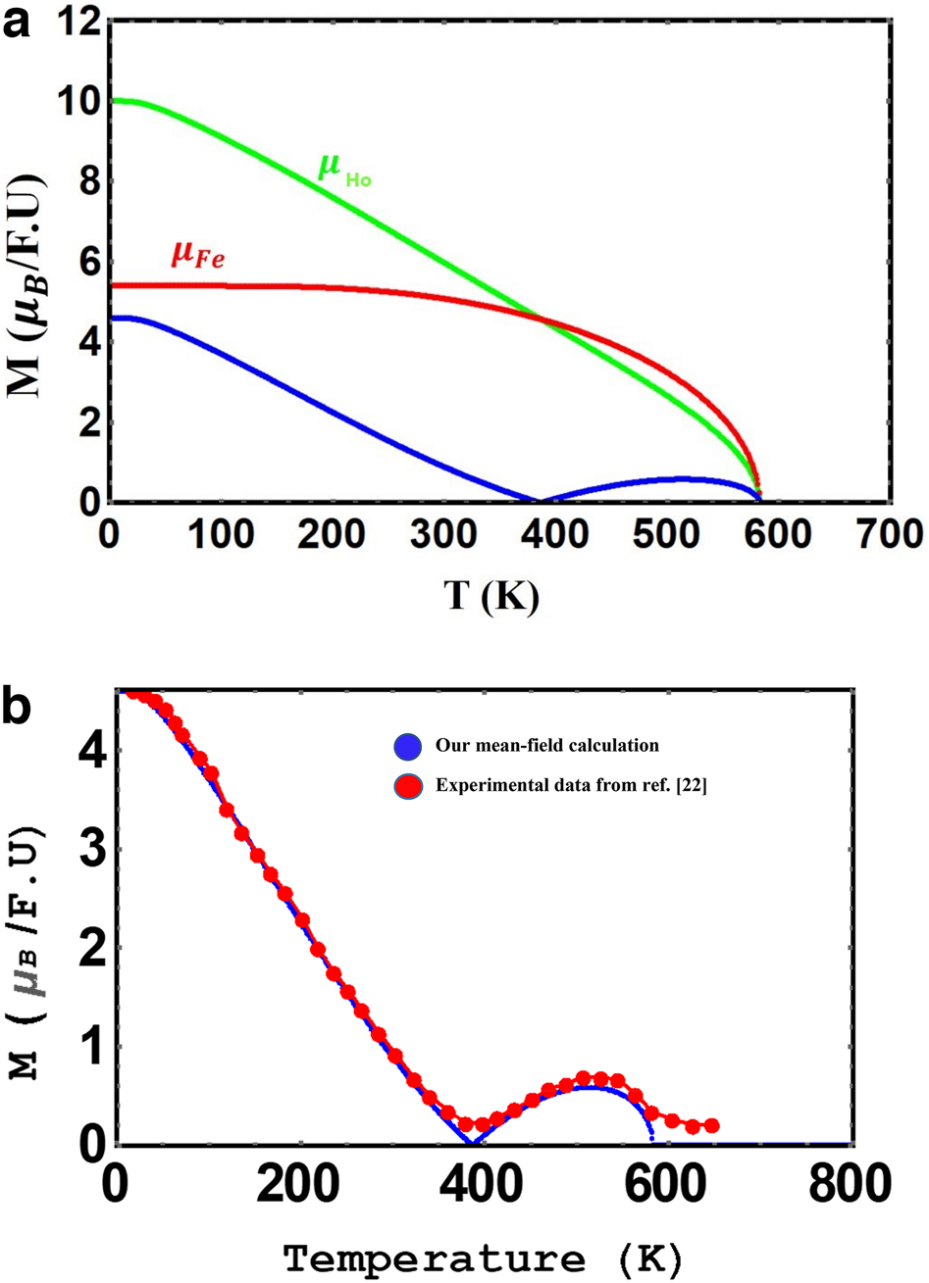
(**a**) Mean-field calculated total and sublattice magnetization of HoFe_3_, in zero field, vs. temperature. (**b**) Total magnetization of HoFe_3_ vs. temperature.

Using the two-sublattice molecular field theory, the temperature dependence of magnetization of the rare earth and Fe sublattices, as well as total magnetization for ${\mathrm{YFe}}_{3}$ and ${\mathrm{HoFe}}_{3}$, are calculated. Total magnetic moments for ${\mathrm{YFe}}_{3}$ and ${\mathrm{HoFe}}_{3}$ ,calculated using the ab initio method are 4.8 µ_B_/f.u and 4.4 µ_B_ /f.u, respectively, which are in good agreement with available experimental values, i.e. 4.88 and 4.59 µ_B_ /f.u as reported by J. F. Herbst et al.^22^. Table 1 displays the magnetic moments calculated in the present work and those by Ref.^22^. Figures 3 and 4a display mean-field- calculated temperature-dependence of magnetization, in zero field for ${\mathrm{YFe}}_{3}$ and ${\mathrm{HoFe}}_{3}\mathrm{respectively}$. The total magnetic moments of these two compounds, at very low temperatures, are in excellent agreement with the values referred to in Table 1. In addition we displayed, in Figs. 3 and 4b, both our mean field calculated magnetization and the experimental data extracted from Ref.^22^.

**Table 1:** Magnetic moments at very low temperature (~ 0 K) using our calculation, in comparison to experimental data.

Method	YFe_3_ (µ_B_)	HoFe_3_ (µ_B_)	Reference
Ab initio	4.8	4.4	Present work
Experimental	4.88	4.59	^22^
Mean-field	4.8	4.6	Present work

Ferromagnetic order for YFe_3_ is evident from Fig. 3 and the magnetic moment per Fe atom is about 1.6 µ_B_ in agreement with the value in Fig. 1 in Herbst work^22^. On the other hand, a ferrimagnetic order with a compensation point is found for HoFe_3_. As the magnetic moments of the Fe and Ho sublattices become equal and antiparallel, a cancellation of the total moment takes place close to 400 K. The continuous decrease in magnetization at Tc is a well-known feature of SOPT materials^54^.

**Figure 5:**
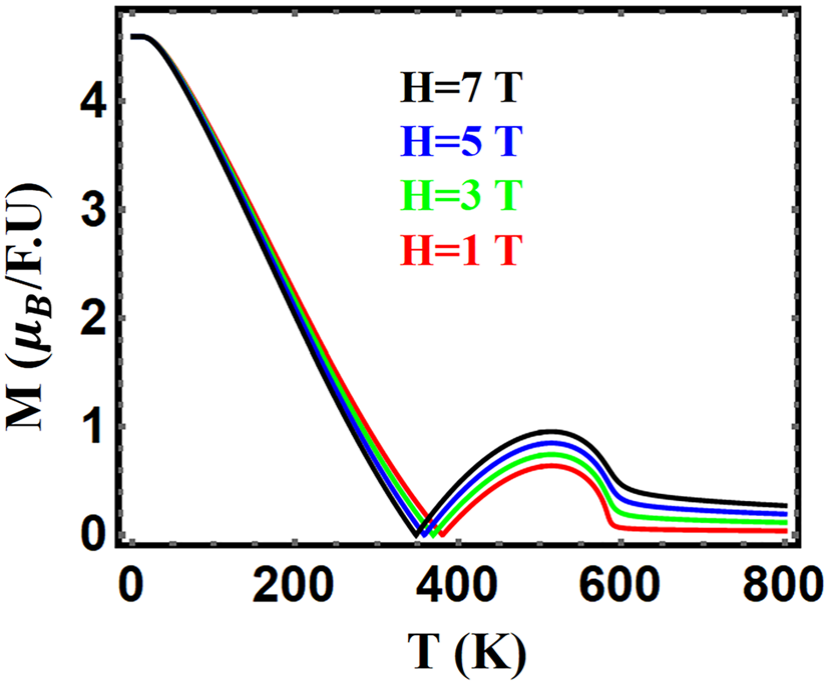
Total magnetization of HoFe_3_ in different applied fields 1, 3, 5 and 7 T vs. temperature.

The temperature dependence of the magnetization, in 1, 3, 5 and 7 T fields, for ${\mathrm{HoFe}}_{3}$, is shown in Fig. 5. an increase in the Curie temperature and a decrease in the compensation temperature is evident, with increasing the field. This behavior is consistent with that found in ferrimagnetic compounds, as reported for example, by P. von Ranke^55^. We may notice that even as the field increases the magnetization still has a continuous drop at Tc. Again, this confirms the SOPT nature of the studied compounds.

### Heat capacity and entropy

**Figure 6:**
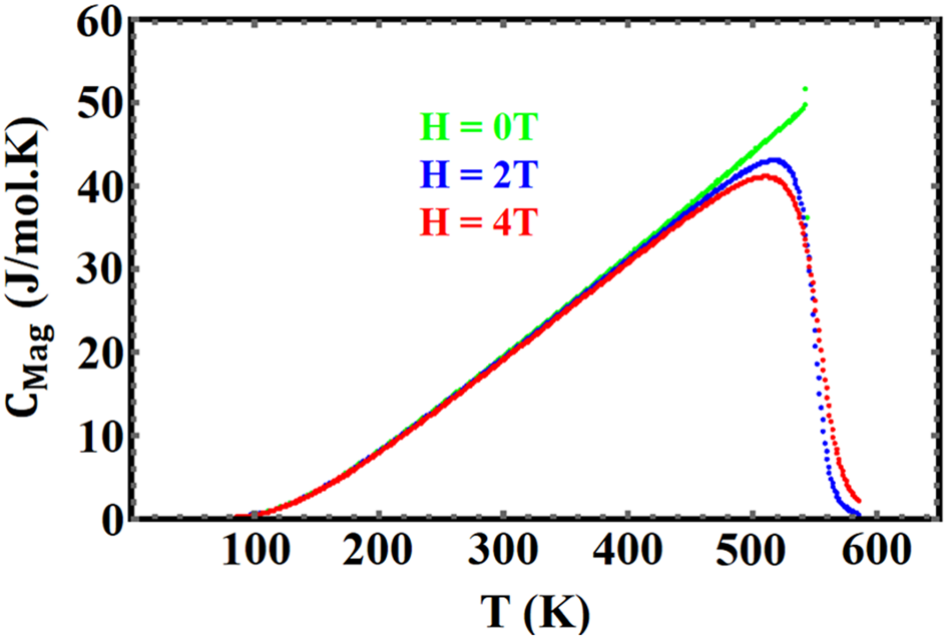
Magnetic heat capacity of ${\mathrm{YFe}}_{3}$ , in different fields, vs. temperature.

**Figure 7:**
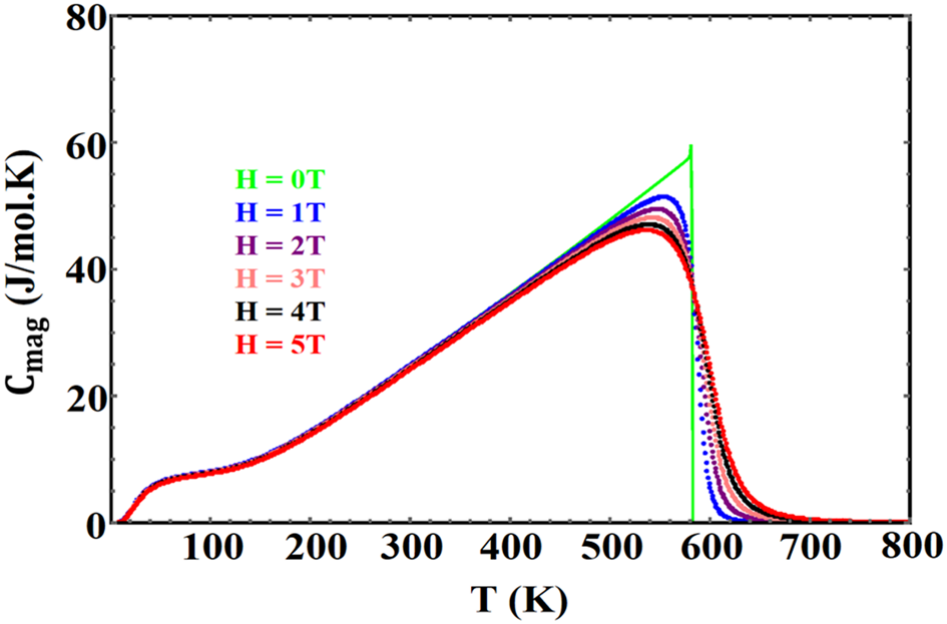
Magnetic heat capacity of HoFe_3_ in different fields, vs. temperature.

As previously stated, three contributions must be calculated to determine the total heat capacity of these materials. Figures 6 and 7 show, respectively, the magnetic heat capacity for ${\mathrm{YFe}}_{3}$ and ${\mathrm{HoFe}}_{3}$ in different applied fields. The features of these two figures, at the Curie temperature, show that the transition is a second order phase transition. In contrast, in the FOPT case, the peak in the heat capacity shifts to higher temperatures as the field increases^53^.

**Figure 8:**
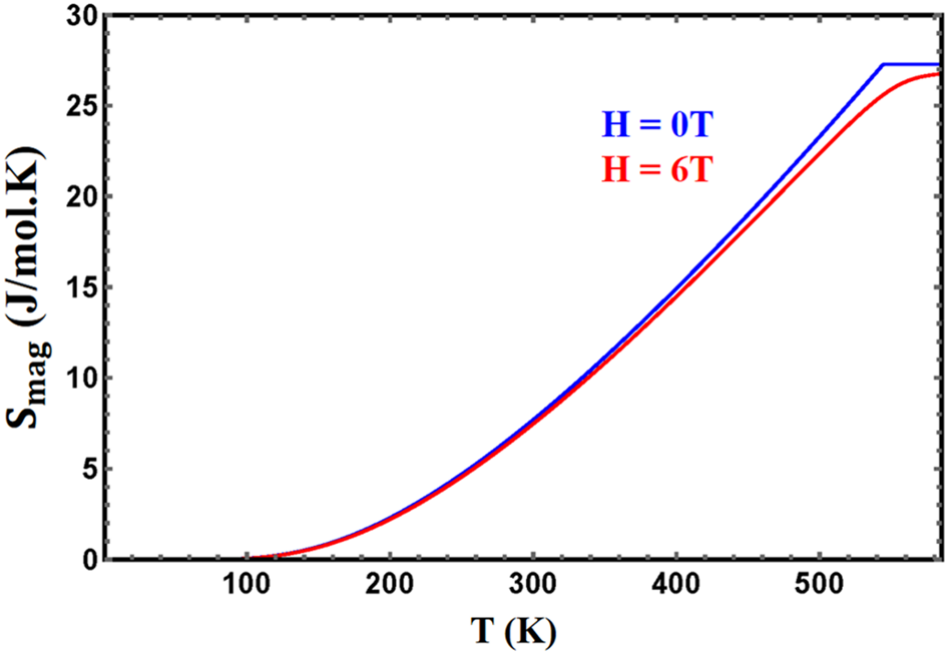
The calculated magnetic entropy of ${\mathrm{YFe}}_{3}$ in two different fields vs. temperature.

**Figure 9:**
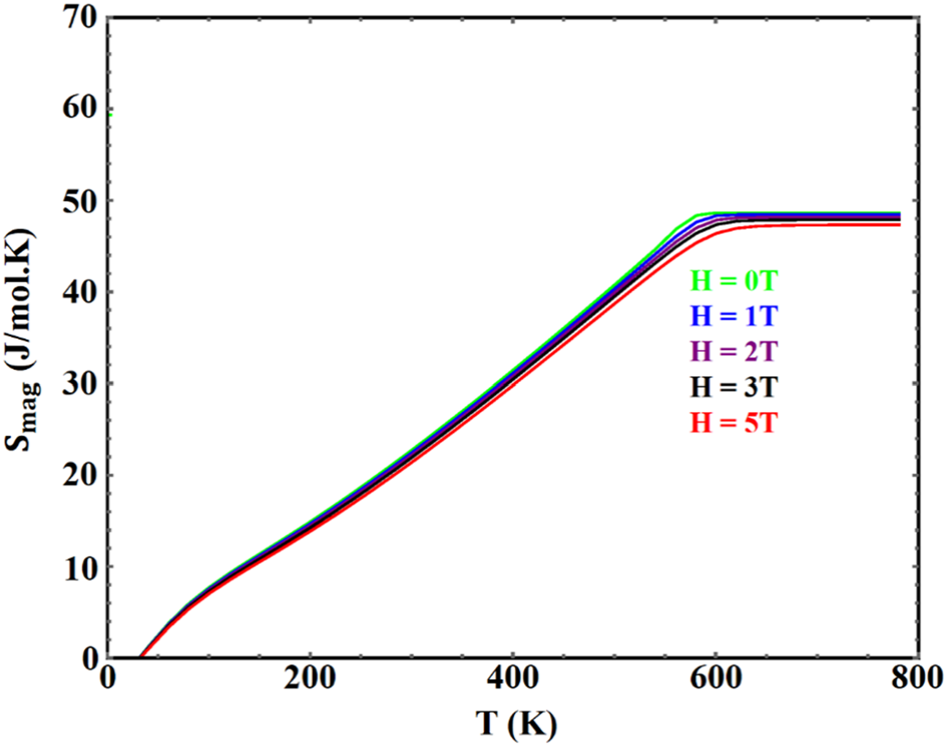
The calculated magnetic entropy of ${\mathrm{HoFe}}_{3}$ in different fields vs. temperature.

The calculated magnetic entropy, in fields in the range 0–6 T, is shown in Figs. 8 and 9 for YFe_3_ and HoFe_3_ respectively. The entropy saturates to its maximum at T_c_ and above, and is reduced by increasing the field. The maximum values of the calculated magnetic entropy are about 27.3 and 50.9 J/mol. K for YFe_3_ and HoFe_3_, respectively. These values are calculated using the equation:

$${\text{S}}_{{{\text{max}}}} = {\text{R }}\left[ {\ln \left( {2{\text{ J}}_{{\text{r}}} + 1} \right) + 3\ln \left( {2{\text{ J}}_{{{\text{Fe}}}} + 1} \right)} \right],$$

where J_r_ and J_Fe_ are the total angular momenta for the rare earth and Fe atoms, respectively. The molecular-field results of the maximum magnetic entropy is in very good agreement with the results of the equation shown above. The gradual increase of the magnetic entropy with temperature until it saturates at T ≥ T_c_ instead of the sudden increase found in FOPT materials show that the transition in these two compounds is a SOPT one.

### Elastic properties of $\mathbf{Y}{\mathbf{F}\mathbf{e}}_{3}$

The elastic properties of YFe_3_ are calculated by using IRelast Package as available in the Wien2k Package^47^, the output of the program is shown in Table 2.

**Table 2:** The Elastic properties of YFe_3_ as obtained from IRelast Package.

a. C_11_, C_12_, C_13_, C_33_, and C_55_ are the five independent elastic constants for a hexagonal symmetry as calculated from IRelast Package			
Final elastic constants of YFe_3_ for a volume = 2466.7026 (Bohr radius)^3^			
C_11_ = 195.3216 GPa C_12_ = 27.0896 GPa C_13_ = 61.1885 GPa C_33_ = 210.5720 GPa C_55_ = 149.9924 GPa			

b. The bulk, shear, and Young modulus along with the Poisson’s coefficient as calculated from IRelast package			
	VOIGT model Prediction	REUSS model Prediction	HILL model Prediction
Bulk modulus	100.016 (GPa)	98.659 (GPa)	99.337 (GPa)
Shear modulus	106.936 (GPa)	95.487 (GPa)	101.211 (GPa)
Young modulus	236.514 (GPa)	216.586 (GPa)	226.655 (GPa)
Poisson's coefficient	0.105	0.134	0.119

c. Transverse, longitudinal, and average elastic sound wave velocity along with the Debye temperature as calculated from IRelast package			
By using HILL data: Transverse elastic wave velocity = 3805.19 (m/s) Longitudinal elastic wave velocity = 5789.42 (m/s) The average wave velocity = 4167.31 (m/s)	Debye temperature = 500.5(K)		

The Debye temperature is calculated from the mean sound velocity. The bulk and shear moduli of ${\mathrm{YFe}}_{3}$, using the Hill model, are 99.337 and 101.211 GPa, respectively. The calculated ${\theta }_{D}$ for ${\mathrm{YFe}}_{3}$ is 500.5 K. For the HoFe_3_ compound, the Debye temperature is 357.85 as reported by the Materials Project site (Kristin Persson)^32^.

### Magnetocaloric effect

#### Isothermal entropy change

**Figure 10:**
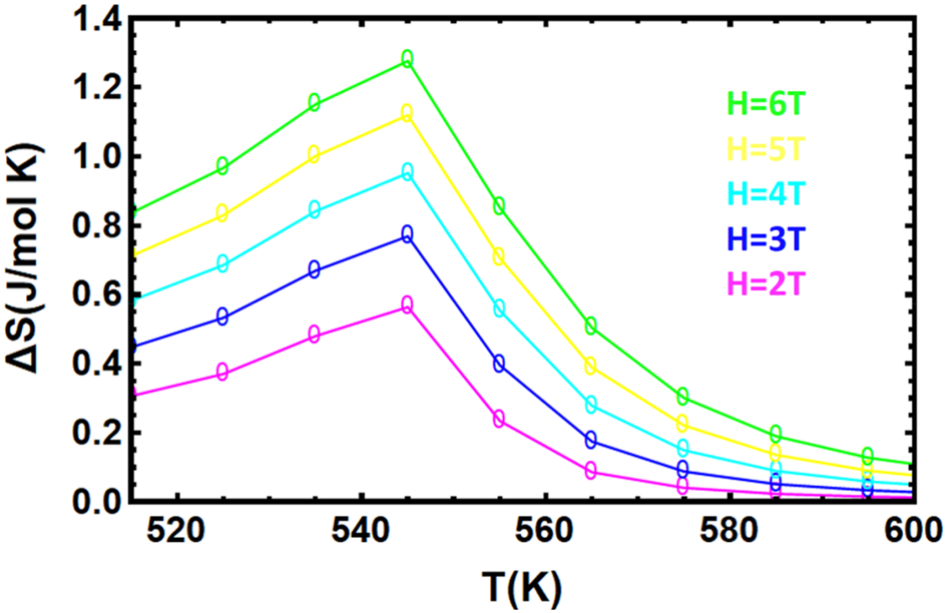
Temperature dependence of isothermal change in entropy for YFe_3_ in fields: 2, 3, 4, 5 and 6 T.

**Figure 11:**
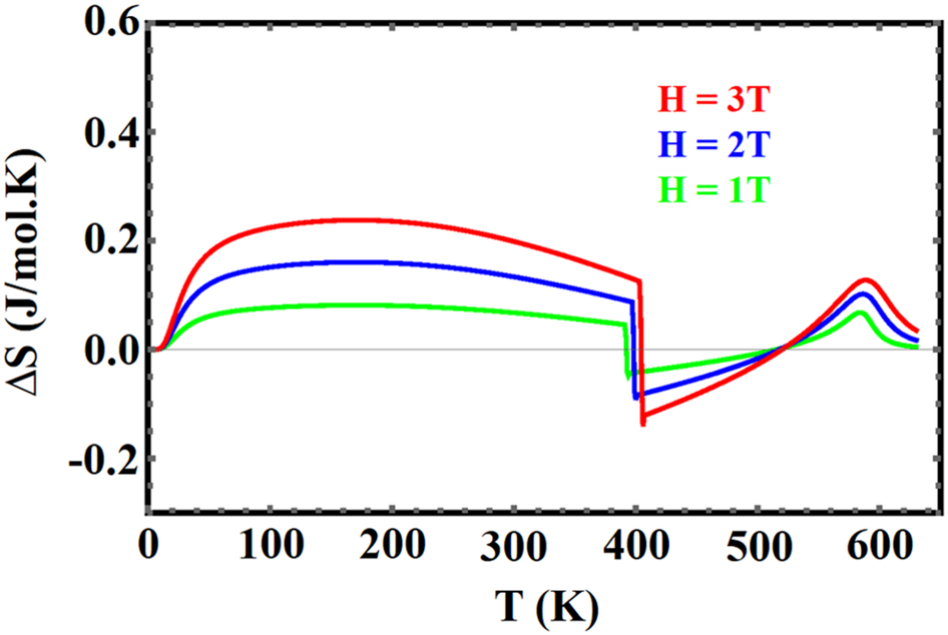
Temperature dependence of isothermal change in entropy for HoFe_3_ in fields of 1, 2 and 3 T.

Figures 10 and 11 show the isothermal entropy change, for different magnetic fields, for ${\mathrm{YFe}}_{3}$ and ${\mathrm{HoFe}}_{3}$ respectively**.** The $\Delta \mathrm{Sm}$ curve for ${\mathrm{HoFe}}_{3}$ exhibits two peaks: the first is a broad peak below the compensation temperature, and the second, smaller peak, has its maximum at the ferrimagnetic-paramagnetic phase transition at ${T}_{C}=590 K$. These two features correspond to the inverse and direct MCE effects, respectively. For ferromagnetic ${\mathrm{YFe}}_{3}$ there is only one peak at a temperature around its T_c_ (545 K). The temperature and field dependences of ∆S_m_ are those of SOPT materials. In particular, the curves at different fields have their maxima at Tc. In FOPT materials, the peak shifts to higher temperatures as the field increases^53^.

#### Adiabatic change in temperature

**Figure 12:**
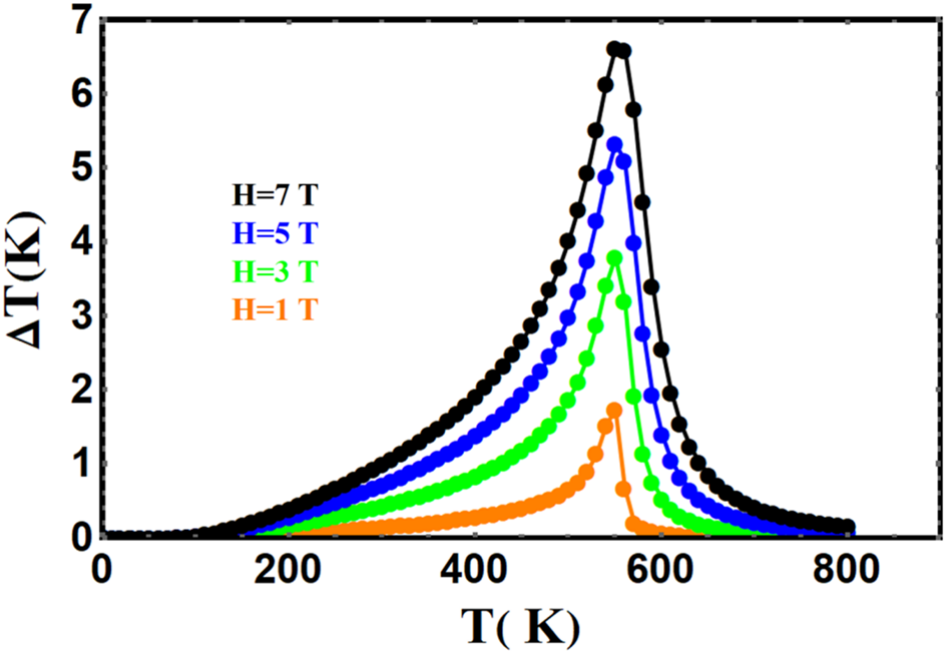
The field-dependence of the adiabatic change in temperature vs. temperature for ${\mathrm{YFe}}_{3}$.

**Figure 13:**
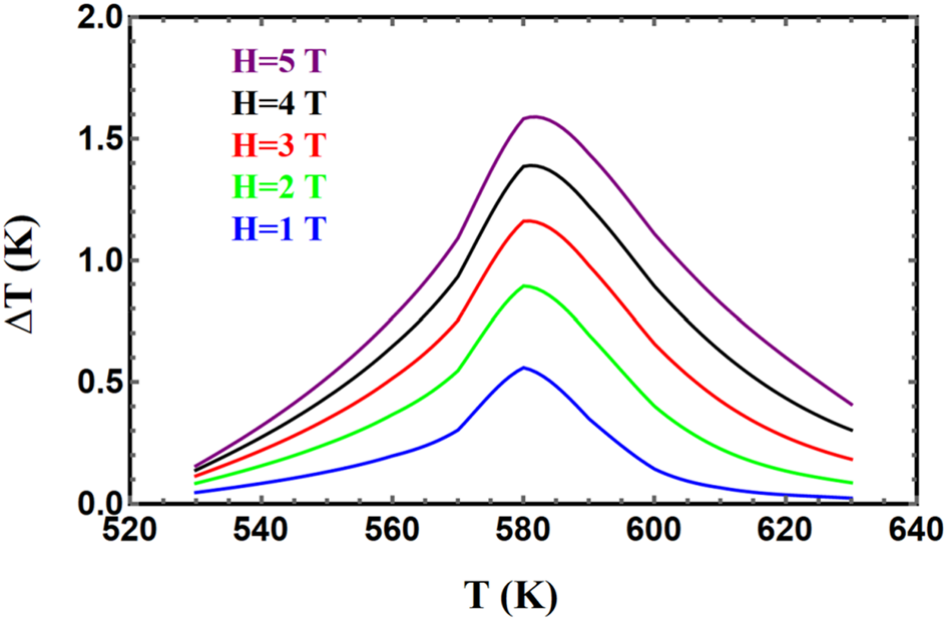
The field-dependence of the adiabatic change in temperature vs. temperature for ${\mathrm{HoFe}}_{3}.$

We have calculated the adiabatic change in temperature for different field changes, as shown in Figs. 12 and 13, for YFe_3_ and HoFe_3_ systems, respectively. It is clear that the former compound has a higher cooling rate than the latter in agreement with reported literature^56^. For example, cooling rates about 1.3 and 0.4 K/T are achieved for these two compounds respectively for a field change of 3 T. The curves in each of Figs. 12 and 13 have their maxima centred at the Curie temperature. This is a known feature of compounds exhibiting second order phase transition and treated via the mean-field theory^53^.

#### The relative cooling power RCP (T)

The RCP is a figure-of-merit for MCE materials (e.g.^56^). It is defined as RCP (T) = ∆T_ad_ (max) × δT_FWHM_. Table 3 displays the RCP (T) for benchmark materials e.g. Gd, Gd-based compounds and FeRh system. From this data, we may conclude that the RCP of YFe_3_ is comparable with those of well-known materials.

**Table 3:** The Relative Cooling Power RCP (T), using our calculation, in comparison to experimental data.

Material	∆H(T)	RCP(T)/∆H (K^2^/T)	Reference
Gd	6	161.2	^56^
Fe	3	95	
Co	2.32	78.4	
Gd_5_Ge_4_	5	50.4	
Gd_5_Si_4_	5	109	
Gd _0.2_ Er _0.8_ Ni Al	5	75.9	
FeRh	2.5	−66.4	
YFe_3_	1.58	34.2	
HoFe_3_	1.58	3.5	
YFe_3_	3	85	Present work
HoFe_3_	3	15	Present work

#### The Arrott plots and universal curve

**Figure 14:**
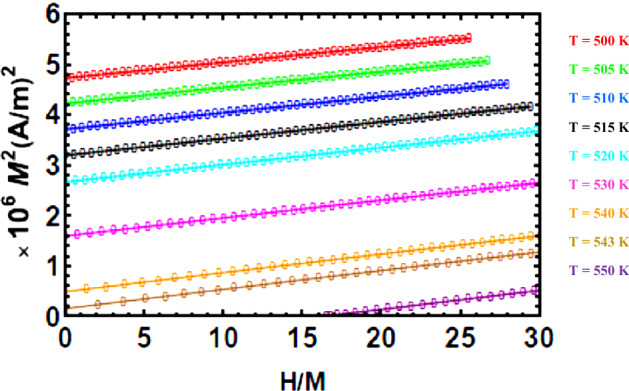
The Arrott plots, at different temperatures, for YFe_3_.

The Arrott plots for YFe_3_ are shown, in a temperature range around T_c_, in Fig. 14. The positive slopes at those temperatures, below and above T_c_, are indicative of second order phase transition. First order transitions exhibit negative or s-shaped slopes^57,58^. The straight line starting near the origin is calculated at a temperature close to Tc (Fig. 3). The features of the universal curve, described below, supports the presence of SOPT as well.

**Figure 15:**
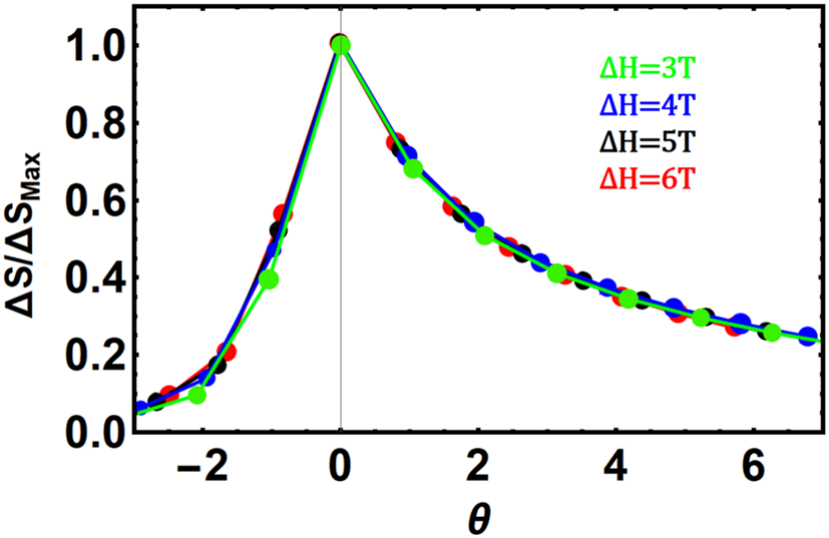
The universal curve for YFe_3_.

The universal curves^59^, for YFe_3_, are shown in Fig. 15 for field changes of 3, 4, 5 and 6 T. The curves are collapsed on each other especially at high temperatures i.e. θ > 0. The parameter θ is given by: θ = (T − T_c_) / (T_r_ − T_c_), where T_r_ is a reference temperature defined as the temperature at which the following condition holds: ∆S_M_ (T_r_) = 0.7 (∆S_M_)^peak^. The collapse of the ΔS_M_ curves is also indicative of second order phase transition, which is different from the features encountered in first order phase transitions^60^.

It would be of interest to support our finding that the mean field theory is suitable for explaining the physical properties. Therefore, calculating some of the critical exponents^61–65^ would be beneficial. We have calculated the exponent n in the relation:

$$\Delta S_{m} \sim H^{n}$$

from the field dependence of ΔS_m_ close to T_c_. The relation between n, β and γ is:

n = 1 + (β−1)/(β + γ) [e.g. ^65^, which can be used to evaluate γ if β is calculated from the relation:

$$M\sim \left( { - t} \right)^{\beta } H = 0, T < T_{c}$$

or calculate β, if γ is calculated from:$\chi_{T} \sim t^{ - \gamma } ,H = 0, T > T_{c}$, where t = (T−T_c_)/T. We have calculated β for YFe_3_ from its M-T data, in zero field, by taking the logarithm of M ~ (−t)^β^ and performing the calculation for temperatures lower than T_c_. We have obtained β around 0.52 for this compound. Therefore, the percentage error between our result and the value 0.5 of the mean-field is around 4%. The factor γ turned out to be around 0.92 for YFe_3_. In addition, we have calculated the critical exponent δ from the relation: δ = 1 + γ/β^66^ and found that δ = 2.8, which is close to the MFT value of 3. From the above analysis, we conclude that mean-field theory has fairly produced the critical exponents for YFe_3_. We would also emphasize the fact that the mean-field theory has its own limitations near T_c_.

We should mention that other models e.g. 3D-Heisnberg, 3D- Ising and Tri-critical mean field model [e.g.^67^] may be used to study the critical exponents, however a future experimental and/or theoretical work, dedicated to this task, may shed more light on the most proper model.

## Conclusions

The two-sublattice molecular field model was used for calculating the thermomagnetic properties for ${\mathrm{YFe}}_{3}$ and ${\mathrm{HoFe}}_{3}$. The temperature dependence of magnetization shows that ${\mathrm{YFe}}_{3}$ is ferromagnetic with a Curie temperature close to 537 K, while ${\mathrm{HoFe}}_{3}$ is a ferrimagnetic compound with a compensation point around 389 K and a Curie temperature close to 565 K. The bulk and shear moduli are calculated using the WIEN2K ab-initio electronic code for YFe_3_ system. Those moduli were used to calculate its Debye temperature (500.53 K). The Debye temperature of HoFe_3_ is obtained from the Materials Project site. Using the calculated DOS, at Fermi energy, the electronic heat capacity coefficient ${\gamma }_{e}$ was found to be 0.0256 and 0.0575 J/${K}^{2}$ mole, for ${\mathrm{YFe}}_{3}$ and ${\mathrm{HoFe}}_{3}$ respectively. The isothermal change in entropy ∆S_m_, for a field change of 3 T, is about 0.8 and 0.12 J/mole. K for these systems, respectively. The Y-system exhibits a larger adiabatic drop in temperature (1.3 K/T), for a 3 T field change, than the Ho-system (0.4 K/T). The relative cooling power RCP(T) of YFe_3_ is comparable to well-known MCE materials The temperature and field dependences of the magnetization, magnetic heat capacity, entropy, the MCE quantities together with the Arrott plots and universal curve, are all indicative of SOPT.

## Data Availability

The datasets generated and/or analyzed during the current study are available from the corresponding author on reasonable request.
